# Supplementary material for: Cost Associated with Adherence to the EAT-Lancet Score in Brazil
Source: Nutrients. 2025 Jan 15;17(2):289. doi: 10.3390/nu17020289 (PMC11767983; doi:10.3390/nu17020289)
Supplement: Supplementary file 1 [file nutrients-17-00289-s001.zip › nutrients-3417374-supplementary.pdf]

nutrients

Supplementary

Table S1: Specific food items included by food group

EAT-Lancet Index	
**Emphasized intake**	
Vegetables	Alfalfa sprouts, Anise, Asparagus, Asparagus chicory, Basil, Bean sprout, Beet greens, Bitter dock, Broccoli, Burdock root, Cabbage, Canned vegetables, Caper, Capiçoba leaf, Carob, Carrot, Carqueja, Caruru, Cauliflower, Celery, Chamomile, Chard, Chayote, Chicory, Chinese cabbage, Cilantro, Clover, Cress, Cucumber, Dried vegetables, Eggplant, Endive, Eucalyptus, Garlic, Garden cress, Ginger, Gourd, Guaco leaves, Kiyo leaves, Leek, Lemon balm, Lemongrass, Lettuce, Malabar spinach, Marjoram, Milkweed, Mint, Mushroom, Mustard, Nigagori plant, Okra, Onion, Ora-pro-nóbis (Brazilian leafy vegetable), Palm heart, Parsley, Pea, Pepper, Pickled vegetables, Pod, Purslane, Pumpkin, Radish, Rosemary, Saffron, Scallion, Sorrel, Spinach, Taioba, Tomato, Turnip, Zucchini.
Fruits	Acai berry, Ajuru fruit, Mari-Mari fruit, Apuruí fruit, Apple, Araça fruit, Arrowroot, Atemoia, Avocado, Bacuri fruit, Banana, Barbados cherry, Biribá fruit, Blackberry, Blueberry, Breadfruit, Cape gooseberry, Cashew, Cherry, Cocoa, Coconut, Coconut Water, Conde fruit, Cranberry, Cupuaçu, Dragon fruit, Dried fruit, Fig, Genipap, Goji berry, Grape, Guama fruit, Guava, Guavira fruit, Hog plum, Jackfruit, Jaboticaba, Jambuaçu, Jurubeba, Kaki, Kiwi, Kumquat, Lemon, Lychee, Mango, Mango Cajá, Mangaba fruit, Mangosteen, Medlar, Melon, Murici, Nectarine, Orange, Papaya, Passion fruit, Peach, Pear, Pineapple, Pitanga, (Brazilian cherry), Pitomba, Pomegranate, Plum, Rambutan, Raspberry, Rose apple, Sapodilla, Seriguela, Souari nut, Soursop, Starfruit, Strawberry, Tamarind, Tangerine, Watermelon, Yellow Uxi.
Whole grains	Almond flour, Apple flour, Banana flour, Barley, Chia seed, Coconut flour, Corn, Cranberry flour, Eggplant flour, Flaxseed, Flaxseed flour, Germ, Gluten, Kibbeh flour, Oat, Oat bran, Oatmeal, Passion fruit flour, Peanut flour, Popcorn, Pumpkin seed, Quinoa, Rice, Rice flour, Wheat, Wheat fiber.
Legumes	Bean, Broad bean, Chickpea, Canned lentils, Lentil, Pigeon pea, Soybeans.
Seafood	Crab, Fish roe, Freshwater fish, Lobster, Mussel, Octopus, Oyster, Saltwater fish, Shellfish, Shrimp, Snail, Squid.
Nuts	Almond, Brazil nut, Cashew nuts, Hazelnut, Macadamia nut, Peanut, Pine nut, Pistachio, Portuguese chestnut, Walnut.
Unsaturated oils	Almond oil, Banana oil, Canola oil, Coconut fat, Coconut oil, Corn oil, Cottonseed oil, Fish oil, Grape seed oil, Olive oil, Palm oil, Peanut oil, Rice oil, Sesame oil, Soybean oil, Sunflower oil, Vegetable shortening

**Limited Intake**	
Beef and lamb	Beef offal, Bison meat, Canned meat, Choice beef, Cured beef, Goat, Jerky, Lamb, Mutton, Prime beef, Select beef, Wild boar.
Pork	Canned pork, Cured and smoked pork, Pork belly, Pork chop, Pork foot, Pork loin, Pork offal, Pork tail, Roast suckling pig.
Poultry	Chicken, Chicken heart, Duck, Mallard duck, Nambu, Poultry offal, Processed chicken, Smoked poultry, Turkey, Quail
Eggs	Alligator egg, Chicken egg, Duck egg, Guinea egg, Quail egg.
Dairy	Buffalo milk, Cheese, Cream, Cream cheese, Cow's milk, Goat's milk, Powdered milk, Skimmed milk, Yogurt.
Potatoes	Brazilian potato, Cassava, Potato, Yam.
Added sugars	Added sugar and sugar present in consumed foods.
